# The effects of platelet‐rich plasma therapy on outcomes after anterior cruciate ligament reconstruction: A systematic review and meta‐analysis

**DOI:** 10.1002/jeo2.70913

**Published:** 2026-09-21

**Authors:** André Bosio Castro, Eiki Kobayashi, Laura Azevedo, Pedro Jorge

**Affiliations:** ^1^ Faculdade de Medicina do ABC Santo André São Paulo Brazil; ^2^ Faculdade de Ciências Médicas da Santa Casa de São Paulo São Paulo São Paulo Brazil

**Keywords:** anterior cruciate ligament, anterior cruciate ligament reconstruction, knee, meta‐analysis, platelet‐rich plasma

## Abstract

**Purpose:**

To determine whether platelet‐rich plasma (PRP) improves outcomes after anterior cruciate ligament reconstruction (ACLR).

**Methods:**

MEDLINE via PubMed, Embase, Scopus, the Cochrane Central Register of Controlled Trials and the Latin American and Caribbean Health Sciences Literature database were searched through 16 July 2026 for comparative studies of PRP in primary ACLR. Outcomes included International Knee Documentation Committee (IKDC) and Lysholm scores, pain measured with a visual analogue scale, instrumented knee stability and magnetic resonance imaging (MRI) findings. Random‐effects meta‐analyses used restricted maximum likelihood estimation with Hartung–Knapp–Sidik–Jonkman adjustments. Post hoc sensitivity analyses included randomized controlled trials only. Certainty of evidence was assessed using the Grading of Recommendations Assessment, Development and Evaluation approach.

**Results:**

Seventeen studies were included. In analyses combining randomized and nonrandomized studies, PRP was associated with small improvements in IKDC scores at 6 months (mean difference [MD], 1.66; 95% confidence interval [CI], 0.31–3.01) and 12 months (MD, 2.37; 95% CI, 1.19–3.54). These differences were no longer statistically significant in randomized‐trial analyses. No robust differences were observed in Lysholm scores at 3, 6 or 12 months. A small late post‐operative Lysholm difference persisted in the randomized‐trial analysis but was based on two trials and was clinically trivial. Pain at approximately 12 months did not differ between groups (MD, −0.60; 95% CI, −1.64 to 0.44). No consistent benefits were demonstrated for objective knee stability or MRI outcomes. Certainty of evidence ranged from very low to low.

**Conclusions:**

PRP does not provide consistent or clinically meaningful benefits after ACLR. Current evidence does not support its routine use.

**Level of Evidence:**

Level II.

**Abbreviations**
ACL: anterior cruciate ligament
ACLR: anterior cruciate ligament reconstruction
BPTB: bone–patellar tendon–bone
BVS: Biblioteca Virtual em Saúde
CENTRAL: Cochrane Central Register of Controlled Trials
CI: confidence interval
CT: computed tomography
GRADE: Grading of Recommendations Assessment
  Development and Evaluation
IKDC: International Knee Documentation Committee
KOOS: Knee injury and Osteoarthritis Outcome Score
KOOS4: mean score of four Knee injury and Osteoarthritis Outcome Score subscales
LILACS: Latin American and Caribbean Health Sciences Literature
MD: mean difference
MRI: magnetic resonance imaging
NR: not reported
PRISMA: Preferred Reporting Items for Systematic Reviews and Meta‐Analyses
PROSPERO: International Prospective Register of Systematic Reviews
PRP: platelet‐rich plasma
RCT: randomized controlled trial
REML: restricted maximum likelihood
ROBINS‐I: Risk Of Bias In Non‐randomized Studies of Interventions
RoB 2: Cochrane Risk of Bias 2 tool
VAS: visual analogue scale
VISA: Victorian Institute of Sport Assessment

## INTRODUCTION

Anterior cruciate ligament (ACL) injuries are among the most common and functionally limiting knee injuries in physically active individuals and athletes, frequently resulting in prolonged absence from sports participation and long‐term functional impairment [27]. Although advances in surgical techniques and rehabilitation protocols have improved clinical outcomes, graft healing and biological integration remain critical determinants of post‐operative recovery, functional performance and long‐term knee stability after ACL reconstruction (ACLR).

In this context, platelet‐rich plasma (PRP) has emerged as a potential biologic adjunct aimed at enhancing the biological environment of graft healing. PRP is an autologous blood‐derived product characterized by a platelet concentration above baseline levels, obtained through centrifugation processes that reduce plasma volume while concentrating platelets. Beyond their role in haemostasis, platelets release a variety of growth factors and cytokines—including platelet‐derived growth factor, transforming growth factor‐β and vascular endothelial growth factor—that are involved in tissue regeneration, angiogenesis and bone–tendon healing [10]. These biological properties provide a mechanistic rationale for the application of PRP in ligament reconstruction procedures.

Over the past decade, PRP has been increasingly adopted in sports medicine and orthopaedic practice, including its use during ACLR, partly influenced by its application in elite athletes and professional sports settings. Experimental studies have suggested that PRP may promote graft remodelling, enhance angiogenesis and improve tendon‐to‐bone integration. Concurrently, clinical investigations have evaluated the effects of PRP on patient‐reported functional outcomes, post‐operative pain, objective knee stability and imaging‐based markers of graft maturation assessed by magnetic resonance imaging (MRI) [15].

Several systematic reviews and meta‐analyses have previously evaluated PRP augmentation in ACLR. Early reviews primarily examined graft maturation, graft–bone interface healing and imaging‐based outcomes, whereas subsequent evidence syntheses incorporated patient‐reported outcomes, pain, objective knee stability and tunnel‐related outcomes. Overall, these reviews have reported inconsistent or small improvements in selected outcomes, without demonstrating a consistent and clinically meaningful benefit in objective stability, graft maturation or long‐term knee function [2, 4, 7, 8, 9, 18, 33, 36].

Accordingly, the rationale for the present review is not the absence of previous evidence syntheses, but the need to determine whether recently published comparative studies modify the existing conclusions. This systematic review updates the evidence through 16 July 2026 and provides time‐specific quantitative analyses of patient‐reported functional outcomes and post‐operative pain, together with an integrated narrative synthesis of objective knee stability and MRI‐based graft assessments. In addition, random‐effects models using restricted maximum likelihood (REML) estimation and Hartung–Knapp–Sidik–Jonkman adjustments were applied to provide more conservative estimates of uncertainty when relatively few studies contributed to each analysis. The certainty of evidence was evaluated using the GRADE approach, and statistically significant effects were interpreted in relation to established thresholds of clinical importance. Therefore, the purpose of this study was to provide an updated and clinically oriented assessment of whether PRP augmentation offers meaningful benefits after ACLR.

## METHODS

### Protocol and registration

This systematic review and meta‐analysis was conducted and reported in accordance with the Preferred Reporting Items for Systematic Reviews and Meta‐Analyses (PRISMA 2020) statement [23]. The review protocol was prospectively registered in the International Prospective Register of Systematic Reviews (PROSPERO; registration number CRD420251109436).

### Study identification and selection

A comprehensive literature search was performed in MEDLINE via PubMed, Scopus, Embase, the Cochrane Central Register of Controlled Trials (CENTRAL) and the Latin American and Caribbean Health Sciences Literature database (LILACS) through the Biblioteca Virtual em Saúde (BVS) interface, from database inception through July 16, 2026. No restrictions were applied regarding publication date or language. The complete search strategies for all databases are presented in Table 1.

**Table 1:** Search strategies used across the electronic databases.

Database	Search strategy	Records retrieved, *n*
PubMed	(‘platelet rich plasma’[MeSH Terms] OR (‘platelet rich’[All Fields] AND ‘plasma’[All Fields]) OR ‘platelet rich plasma’[All Fields] OR (‘platelet’[All Fields] AND ‘rich’[All Fields] AND ‘plasma’[All Fields]) OR ‘platelet rich plasma’[All Fields]) AND (‘anterior cruciate ligament’[MeSH Terms] OR (‘anterior’[All Fields] AND ‘cruciate’[All Fields] AND ‘ligament’[All Fields]) OR ‘anterior cruciate ligament’[All Fields] OR ‘acl’[All Fields] OR (‘anterior cruciate ligament’[MeSH Terms] OR (‘anterior’[All Fields] AND ‘cruciate’[All Fields] AND ‘ligament’[All Fields]) OR ‘anterior cruciate ligament’[All Fields]))	245
Scopus	TITLE‐ABS‐KEY (platelet‐rich plasma AND (ACL OR anterior cruciate ligament))	246
Embase	(‘platelet‐rich plasma’/exp OR ‘platelet‐rich plasma’ OR (‘platelet rich’ AND (‘plasma’/exp OR plasma))) AND (‘acl’/exp OR acl OR ‘anterior cruciate ligament’/exp OR ‘anterior cruciate ligament’ OR (anterior AND cruciate AND (‘ligament’/exp OR ligament)))	496
LILACS via BVS	platelet‐rich plasma AND (acl OR anterior cruciate ligament) AND (db:(‘LILACS’))	244
CENTRAL	(MeSH descriptor: [Platelet‐Rich Plasma] explode all trees OR (‘platelet rich plasma’ OR ‘platelet‐rich plasma’ OR PRP):ti,ab,kw) AND (MeSH descriptor: [Anterior Cruciate Ligament] explode all trees OR (‘anterior cruciate ligament’ OR ACL OR ACLR):ti,ab,kw)	75

*Note*: Searches were updated through 16 July 2026. Strategies and record counts were reproduced from the authors' search log.

All retrieved records were imported into a Microsoft Excel spreadsheet (Microsoft Corporation) for screening and management. Duplicate records were identified and removed before title and abstract screening. Title and abstract screening was performed independently by two reviewers (A.B.C. and E.K.). Full‐text articles of potentially eligible studies were subsequently assessed independently by the same reviewers according to the predefined eligibility criteria. Discrepancies at any stage were resolved through discussion and consensus. When necessary, a third reviewer (P.J.) was consulted to reach a final decision.

### Eligibility Criteria

Studies were included if they met the following criteria:

- *Population*: Predominantly adult populations undergoing primary ACLR. Studies enroling mixed adolescent and adult populations were eligible when the cohort was predominantly adult and age‐stratified outcome data were unavailable.
- *Intervention*: Adjunctive use of PRP, administered intraoperatively and/or post‐operatively.
- *Comparator*: ACLR performed without PRP.
- *Outcomes*: Patient‐reported functional outcomes (International Knee Documentation Committee [IKDC] score, Lysholm score, Knee injury and Osteoarthritis Outcome Score [KOOS], Tegner activity scale), pain assessed using the visual analogue scale (VAS), objective knee stability measured with the KT‐1000 arthrometer and imaging outcomes assessed by magnetic resonance imaging (MRI).

Both randomized controlled trials (RCTs) and nonrandomized comparative clinical studies were eligible for inclusion.

### Data extraction

Data extraction was performed by one investigator (A.B.C.) using a standardized data collection form. A second investigator (E.K.) subsequently verified the extracted data for accuracy and completeness. Discrepancies identified during verification were resolved through discussion and consensus. Extracted data included study design, sample size, patient age and baseline characteristics, graft type, PRP preparation system, centrifugation protocol, whole‐blood volume processed, final PRP volume administered, platelet concentration or fold increase, leucocyte content, activation method, timing and site of PRP application, number of applications, PRP storage conditions when reported, follow‐up duration, enrolment period and reported primary and secondary outcomes. When relevant data were missing or unclear, corresponding authors were contacted to request additional information.

### Risk of bias (RoB) assessment

The RoB of the included studies was independently assessed by two reviewers (A.B.C. and E.K.), with disagreements resolved through discussion and, when necessary, consultation with a third reviewer (P.J.). RCTs were evaluated using the Cochrane Risk of Bias 2 (RoB 2) tool for the effect of assignment to the intervention [13]. The five RoB 2 domains included bias arising from the randomization process, deviations from intended interventions, missing outcome data, measurement of the outcome and selection of the reported result. Domain‐level and overall judgements were classified as low RoB, some concerns or high RoB.

Nonrandomized comparative studies were evaluated using the 2016 version of the Risk Of Bias In Non‐randomized Studies of Interventions (ROBINS‐I) tool [30]. The seven domains included bias due to confounding, selection of participants, classification of interventions, deviations from intended interventions, missing data, measurement of outcomes and selection of the reported result. Judgements were classified as low, moderate, serious or critical RoB or no information.

Because RoB 2 and ROBINS‐I are outcome‐specific instruments, a principal outcome or result was selected for each study assessment. For studies reporting multiple outcomes, priority was given to an outcome contributing directly to the review synthesis or to the study's primary outcome. The results of RoB 2 and ROBINS‐I were presented separately because their domains and judgement categories are not directly interchangeable.

### Certainty of evidence assessment

The certainty of evidence for each outcome was assessed using the Grading of Recommendations Assessment, Development and Evaluation (GRADE) approach [12]. This evaluation considered RoB, inconsistency, indirectness, imprecision and potential publication bias. The overall certainty of evidence was rated as high, moderate, low or very low according to GRADE guidance. Assessments were conducted independently by two reviewers (A.B.C. and E.K.), with disagreements resolved by consensus. For outcomes supported by both randomized and nonrandomized evidence, GRADE ratings were based on the sensitivity analyses restricted to RCTs. The VAS analysis already included RCTs only. Imprecision was judged using prespecified thresholds for clinical importance of 9 points for the IKDC score, 10 points for the Lysholm score and 1.0 point for pain measured on a 0–10 VAS [21, 22].

### Data analysis

Statistical analyses were performed using RevMan Web software (Cochrane Collaboration). A total of eight primary meta‐analyses were conducted: four evaluating the Lysholm score at 3 months, 6 months, 12 months and late post‐operative follow‐up; three evaluating the IKDC subjective score at 3, 6 and 12 months; and one evaluating pain assessed using the VAS at approximately 12 months.

As all outcomes were reported as continuous variables using identical measurement scales across studies, pooled effects were calculated as mean differences (MDs) with corresponding 95% confidence intervals (CIs). Meta‐analyses were performed using the generic inverse variance method, which weights studies according to the precision of their estimates.

Random‐effects models were applied for all analyses to account for between‐study variability. Between‐study variance (*τ* ^2^) was estimated using the REML method, and CIs were adjusted using the Hartung–Knapp–Sidik–Jonkman method, given its improved performance in meta‐analyses with a limited number of studies [25].

Statistical heterogeneity was assessed using the *I* ^2^ statistic and *τ* ^2^ estimates, with *I* ^2^ values interpreted according to conventional thresholds for low, moderate and high heterogeneity.

Post hoc sensitivity analyses restricted to RCTs were performed for all IKDC and Lysholm outcomes to evaluate the robustness of the pooled estimates according to study design. Nonrandomized comparative studies were excluded from these analyses. A separate sensitivity analysis was not required for the VAS outcome because all studies contributing to that meta‐analysis were RCTs. The sensitivity analyses used the same effect measure and statistical methods as the primary analyses, including MDs, random‐effects models, REML estimation of between‐study variance and Hartung–Knapp–Sidik–Jonkman adjustments.

Exploratory subgroup analyses according to PRP formulation, leucocyte content, activation method, graft type and application site were considered. However, these analyses were not performed because PRP characteristics were incompletely and inconsistently reported and because only three to five studies contributed to each pooled outcome. Further stratification would therefore have resulted in very small and clinically heterogeneous subgroups, producing unreliable estimates. PRP preparation and application characteristics were instead summarized descriptively at the study level.

Formal assessment of publication bias (e.g., funnel plot asymmetry or statistical tests) was not performed because the number of studies included in each meta‐analysis was insufficient for reliable evaluation.

All analyses were conducted independently by two investigators (A.B.C. and E.K.). Any discrepancies in data extraction, statistical analysis or interpretation of results were resolved through discussion and consensus.

### Use of artificial intelligence‐assisted technology

During the preparation and revision of this manuscript, the authors used OpenAI's ChatGPT to assist with English‐language editing, organization, clarity and the preparation of responses to reviewers. The tool was not used for study selection, data extraction, RoB assessment, certainty‐of‐evidence assessment or statistical analysis. All generated content was critically reviewed, verified and edited by the authors, who take full responsibility for the accuracy and integrity of the final manuscript.

### Ethical considerations

This study did not involve direct interaction with human participants or access to identifiable patient data and therefore did not require institutional review board approval. All included studies involved human participants, and ethical approval or oversight was reported in the original publications when required. The date range for patient enrolment and/or treatment was extracted from each primary study and is presented in Table 2.

**Table 2:** Characteristics of the included studies.

Panel A. Study design and participant characteristics				
Study	Study design	Enrolment period	Sample size, PRP/control	Graft type
Ji et al. [14]	Randomized controlled trial	Aug 2014–Aug 2016	21/21 randomized; 17/19 analyzed	Autologous semitendinosus–gracilis hamstring
Qureshi et al. [24]	Prospective quasi‐experimental comparative study (odd–even allocation)	Oct 2022–Sep 2023	20/20	Autologous bone–patellar tendon–bone
Cervellin et al. [5]	Prospective randomized controlled trial	2008–2009	20/20	Autologous bone–patellar tendon–bone
Gong et al. [11]	Prospective randomized controlled trial	NR	30/30 randomized; 27/26 at 12 mo	Autologous four‐strand semitendinosus–gracilis hamstring
Walters et al. [34]	Prospective double‐blind randomized controlled trial	2011–2015	27/23 analyzed; 59 randomized before intraoperative exclusions	Autologous bone–patellar tendon–bone
Chen et al. [6]	Retrospective comparative cohort study	Aug 2017–Jun 2019	42/43	Autologous semitendinosus–gracilis hamstring
Rupreht et al. [26]	Randomized controlled imaging trial	NR	25/25	Autologous double‐looped semitendinosus–gracilis hamstring
Valentí Nin et al. [32]	Prospective randomized double‐blind controlled trial	NR	50/50	Bone–patellar tendon–bone allograft
Ye et al. [35]	Surgeon‐ and investigator‐masked randomized clinical trial	21 Mar 2021–18 Aug 2022	60/60 randomized; 114/120 available for primary outcome	Autologous semitendinosus–gracilis grafts for double‐bundle ACLR
Munde et al. [20]	Single‐blind prospective randomized controlled trial	Nov 2018–Feb 2020	44/43 randomized; 40/40 analyzed	Autologous quadrupled semitendinosus–gracilis hamstring
de Almeida et al. [1]	Prospective randomized controlled trial	Nov 2008–Feb 2010	12/15 randomized	Autologous bone–patellar tendon–bone
Magnussen et al. [17]	Matched historical cohort study	PRP: Jan 2007–Aug 2009; control: Jan 2003–Dec 2006	50/50 matched; 29 pairs with 2‐y patient‐reported outcomes	Tibialis tendon allograft
Vadalà et al. [31]	Prospective randomized controlled trial	NR	20/20	Autologous hamstring
Kumar et al. [16]	Prospective randomized controlled trial	NR	35/35	Autologous quadrupled hamstring
Sözkesen et al. [28]	Prospective nonrandomized comparative study (chronological groups)	Mar 2014–Jul 2015	18/26	Autologous four‐strand hamstring
Mirzatolooei et al. [19]	Prospective randomized clinical trial	Feb 2011–Feb 2012	25/25 randomized; 23/23 at 3 mo	Autologous quadrupled hamstring
Starantzis et al. [29]	Prospective double‐blind randomized study	Dec 2007–Jun 2010	30/30 randomized; 25/26 completed	Autologous quadrupled semitendinosus–gracilis hamstring

Panel B. PRP intervention, follow‐up and outcomes			
Study	PRP intervention and application site	Follow‐up	Reported outcomes
Ji et al. [14]	Graft soaked in PRP with intra‐articular PRP at surgery; repeat intra‐articular PRP on post‐operative Days 15 and 30	3 and 12 mo; MRI and second‐look arthroscopy at 12 mo	VAS, Lysholm, IKDC; MRI; second‐look arthroscopy
Qureshi et al. [24]	Activated PRP gel coated the ligamentous graft, was sutured within the graft and was placed in the femoral and tibial tunnels	3 and 6 mo; mean follow‐up 8 mo (range: 6–10)	IKDC, Lysholm; MRI graft maturation
Cervellin et al. [5]	PRP gel applied to the patellar tendon donor defect and the patellar and tibial bone harvest sites	12 mo	VAS, VISA; MRI donor‐site healing
Gong et al. [11]	PRP‐filled gelatin sponge secured within the graft; remaining PRP injected into the graft and bone tunnels	Up to 12 mo; CT at 4 d and 12 mo; MRI at 12 mo	IKDC, Lysholm, Tegner; CT tunnel diameters; MRI signal‐to‐noise quotient
Walters et al. [34]	Activated PRP mixed with autologous bone chips and placed in the patellar donor defect; sham control	12 wk, 6 mo, 1 y and 2 y; MRI at 6 mo	Kneeling pain, pain with activities of daily living, IKDC; MRI donor‐site healing
Chen et al. [6]	PRP injected into the femoral and tibial graft ends; graft soaked in remaining PRP; residual PRP applied around graft ends	3, 6 and 12 mo	IKDC, Lysholm; MRI signal‐to‐noise quotient
Rupreht et al. [26]	PRP gel applied to the graft and into the femoral and tibial tunnels after graft positioning	1, 2.5 and 6 mo	Diffusion‐weighted and dynamic contrast‐enhanced MRI of tibial‐tunnel oedema and vascularity
Valentí Nin et al. [32]	Platelet‐enriched gel placed within and around the allograft and in the tibial tunnel	Mean 24 mo	VAS, IKDC, KT‐1000; MRI graft appearance; inflammatory parameters
Ye et al. [35]	Three post‐operative ultrasound‐guided intra‐articular PRP injections at 4 wk, 8 wk and 3 mo	4 and 8 wk; 3, 6 and 12 mo	KOOS4, IKDC, Lysholm, Tegner, physical examination; MRI graft maturity; adverse events
Munde et al. [20]	PRP injected into the femoral tunnel immediately before graft passage	6 mo	Lysholm, range of motion, clinical stability tests; MRI graft and tunnel healing
de Almeida et al. [1]	PRP gel completely filled the patellar tendon harvest‐site defect	6 mo	MRI patellar‐tendon gap area; VAS, knee questionnaires, isokinetic testing
Magnussen et al. [17]	Intra‐articular portion of the allograft coated with PRP after graft fixation	10 d, 8 wk and 2 y	KOOS, IKDC; effusion, reoperation and graft failure
Vadalà et al. [31]	Liquid and semisolid PRP applied at the femoral and tibial tunnel–graft interfaces	Median 14.7 mo (range 10–16)	CT tunnel widening; IKDC, Lysholm, Tegner, KT‐1000
Kumar et al. [16]	PRP injected into the femoral and tibial tunnels, graft substance and intra‐articularly	Immediate post‐operative, 6 wk and 12 wk	Lysholm, anterior drawer and Lachman tests; tibial‐tunnel widening
Sözkesen et al. [28]	Graft soaked in PRP and PRP injected into the femoral and tibial tunnels	CT on post‐operative Day 1 and at 3 mo; median clinical follow‐up 12 mo (range: 4–16)	CT tunnel widening; IKDC, Lysholm, Tegner, KT‐1000
Mirzatolooei et al. [19]	Graft immersed in PRP; PRP injected into the femoral and tibial tunnels	Post‐operative Day 1 and 3 mo	CT tunnel widening; KT‐1000 and clinical examination
Starantzis et al. [29]	PRP placed between graft strands before passage and injected into the femoral tunnel after fixation	Early post‐operative and ≥12‐mo MRI; mean 14.2 mo (range: 11.2–37)	MRI femoral‐tunnel widening; Lysholm, Tegner, Rolimeter, pivot‐shift test

*Note*: All control groups underwent ACLR without PRP augmentation unless otherwise specified. Sample sizes are presented as PRP/control and refer to the analyzed population when available. NR indicates not reported.

Abbreviations: ACLR, anterior cruciate ligament reconstruction; BPTB, bone–patellar tendon–bone; CT, computed tomography; IKDC, International Knee Documentation Committee; KOOS, Knee injury and Osteoarthritis Outcome Score; MRI, magnetic resonance imaging; NR, not reported; PRP, platelet‐rich plasma; VAS, visual analogue scale; VISA, Victorian Institute of Sport Assessment.

## RESULTS

### Study selection

**Figure 1:**
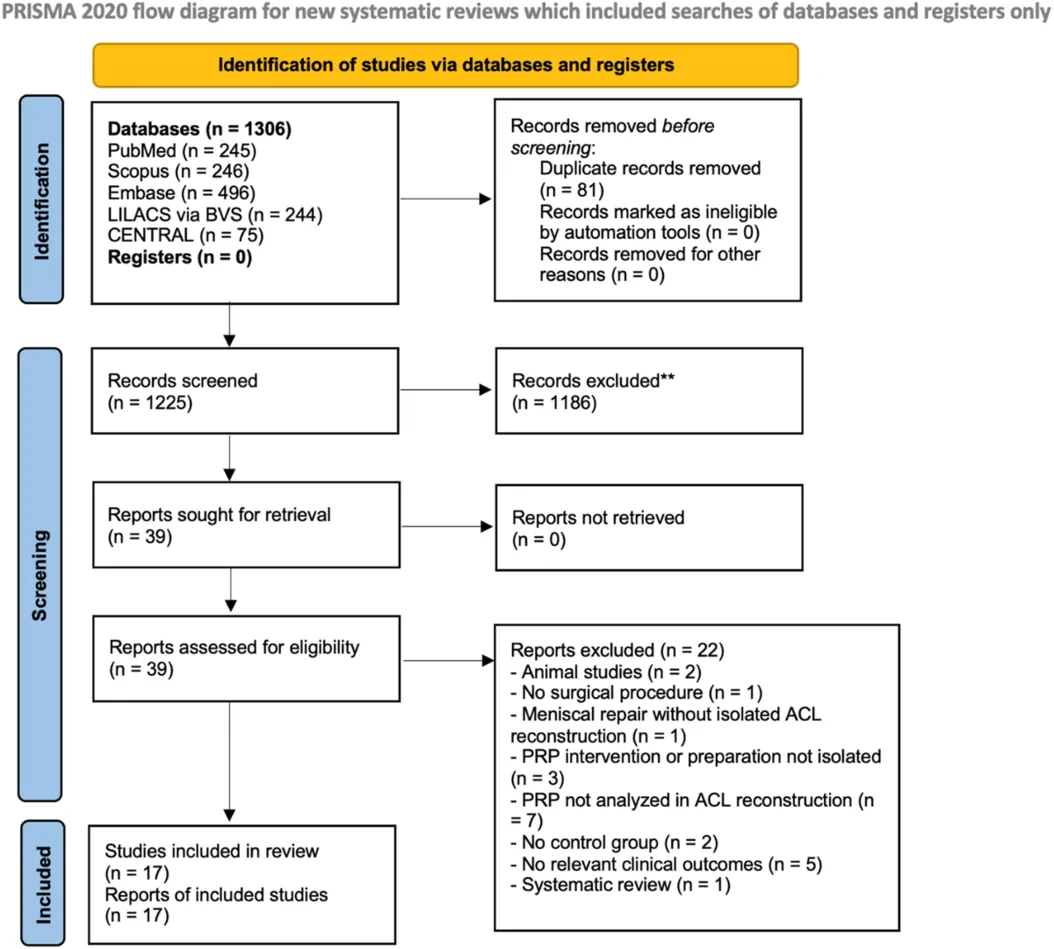
PRISMA 2020 flow diagram of the study selection process. ACL, anterior cruciate ligament; PRP, platelet‐rich plasma.

The updated literature search across MEDLINE via PubMed, Scopus, Embase, the Cochrane Central Register of Controlled Trials (CENTRAL) and LILACS via the Biblioteca Virtual em Saúde identified 1306 records: 245 from PubMed, 246 from Scopus, 496 from Embase, 244 from LILACS and 75 from CENTRAL. After removal of 81 duplicate records, 1225 unique records underwent title and abstract screening, of which 1186 were excluded. Thirty‐nine reports were sought for retrieval; all were successfully retrieved and assessed for eligibility. Of these, 22 were excluded for the following reasons: animal studies (*n* = 2), absence of a surgical procedure (*n* = 1), inclusion of meniscal repair without isolated ACLR (*n* = 1), failure to isolate the PRP intervention (*n* = 3), lack of analysis of PRP in ACLR (*n* = 7), absence of a control group (*n* = 2), absence of relevant clinical outcomes (*n* = 5) and systematic review design (*n* = 1). Ultimately, 17 studies met the eligibility criteria and were included in the systematic review and meta‐analysis. The study selection process is summarized in Figure 1.

### Study characteristics

The characteristics of the included studies are summarized in Table 2. A total of 17 comparative clinical studies evaluating PRP as an adjunct to ACLR were included [1, 3, 5, 6, 11, 14, 16, 17, 19, 20, 24, 26, 28, 29, 31, 34, 35]. Study designs comprised RCTs and comparative observational studies, enroling predominantly young and physically active adults undergoing primary ACLR using hamstring tendon, patellar tendon or allograft grafts.

PRP was administered intraoperatively, post‐operatively or using combined approaches depending on individual study protocols. Substantial heterogeneity was observed in PRP preparation and application, including centrifugation techniques, platelet concentration, activation methods, injected volume and site of application (intra‐articular, graft surface or bone tunnels). Control groups underwent standard ACLR without PRP augmentation.

Follow‐up duration varied across studies, ranging from early post‐operative assessments to follow‐up exceeding 12 months. The enrolment period of the included studies ranged from January 2003 to February 2020; however, several studies did not explicitly report recruitment dates. The most frequently reported outcomes included patient‐reported functional scores (IKDC and Lysholm), post‐operative pain assessed using the VAS, objective knee stability measured with the KT‐1000 arthrometer, activity level assessed using the Tegner scale, knee‐related quality of life assessed using the KOOS and imaging‐based outcomes evaluated using MRI.

### PRP preparation and application characteristics

Reporting of PRP preparation and application characteristics was incomplete and heterogeneous across the included studies. Graft type and application site were generally reported, whereas platelet concentration, leucocyte content and activation method were frequently absent or insufficiently described. Preparation systems, centrifugation protocols, administered volumes, timing and anatomical sites of application also varied substantially. In several reports, it was unclear whether the stated volume represented the whole‐blood volume processed or the final PRP volume administered. Given the limited number of studies contributing to each outcome and the incomplete reporting of these variables, reliable subgroup analyses could not be performed. Detailed study‐level PRP characteristics and the availability of each variable are presented in Table S1.

### Overall critical appraisal

Across the included studies, considerable heterogeneity was observed in study design, PRP preparation and delivery, graft selection, follow‐up duration and outcome reporting. RCTs generally provided higher methodological rigour but were frequently limited by small sample sizes, incomplete reporting of PRP composition (e.g., platelet and leucocyte concentration, activation methods) and variability in application strategies (intra‐articular injection vs. graft or tunnel augmentation).

Comparative observational studies contributed clinically relevant real‐world data but were inherently more susceptible to confounding related to baseline activity level, concomitant procedures, rehabilitation adherence and graft choice, thereby limiting causal inference regarding PRP effectiveness.

Imaging‐based endpoints further contributed to inconsistency, as MRI protocols varied substantially with respect to sequence selection, regions of interest, scoring systems and timing of assessment. Consequently, imaging findings were difficult to compare directly and did not consistently parallel patient‐reported or functional outcomes.

### RoB assessment

RoB assessments were presented separately according to study design and assessment tool. Thirteen RCTs were evaluated using RoB 2, whereas four nonrandomized comparative studies were evaluated using ROBINS‐I. Domain‐level RoB 2 judgements are presented in Table 3, and ROBINS‐I judgements are presented in Table 4. The corresponding visual summaries are presented in Tables 5 and 6, respectively. Complete signalling‐question assessments are provided in Tables S2 and S3.

**Table 3:** RoB 2 domain‐level judgements for principal outcomes of randomized controlled trials.

RoB 2 judgements are result‐specific. The principal outcome assessed for each trial is explicitly identified below; these judgements should not be interpreted as applying automatically to every outcome reported by the study.							
Study	Outcome/result assessed	D1	D2	D3	D4	D5	Overall
Ji et al.	IKDC score at 12 months	Some concerns	Low risk	Some concerns	High risk	Some concerns	High risk
Cervellin et al.	VAS donor‐site pain at 12 months	Some concerns	Low risk	Low risk	Low risk	Some concerns	Some concerns
Gong et al.	IKDC score at 12 months	Some concerns	Low risk	Low risk	High risk	Some concerns	High risk
Walters et al.	Kneeling pain at 6 months	Low risk	Some concerns	Low risk	Low risk	Low risk	Some concerns
Rupreht et al.	MRI tibial‐tunnel parameters at 6 months	Some concerns	Low risk	Some concerns	Low risk	Some concerns	Some concerns
Valentí Nin et al.	MRI graft appearance at 24 months	Some concerns	Low risk	Low risk	Low risk	Some concerns	Some concerns
Ye et al.	KOOS4 at 12 months (primary outcome)	Low risk	Low risk	Low risk	High risk	Low risk	High risk
de Almeida et al.	MRI patellar‐tendon gap area at 6 months (primary outcome)	Some concerns	Low risk	Some concerns	Low risk	Some concerns	Some concerns
Vadalà et al.	CT‐assessed tunnel widening at final follow‐up	Some concerns	Low risk	Low risk	Low risk	Some concerns	Some concerns
Kumar et al.	Lysholm score at 12 weeks	Some concerns	Low risk	Low risk	High risk	Some concerns	High risk
Mirzatolooei et al.	CT‐assessed tunnel widening at 3 months	Some concerns	Low risk	Low risk	Some concerns	Some concerns	Some concerns
Starantzis et al.	MRI‐assessed femoral‐tunnel widening at ≥12 months	Some concerns	Some concerns	Some concerns	Low risk	Some concerns	Some concerns
Munde et al.	MRI graft healing at 6 months (principal radiological outcome)	Low risk	Low risk	Low risk	Low risk	Low risk	Low risk
*D1: randomization process; D2: deviations from intended interventions; D3: missing outcome data; D4: measurement of the outcome; D5: selection of the reported result*.							
Judgement categories: Low risk, Some concerns or High risk.							

*Note*: Green indicates low risk of bias, yellow indicates some concerns, and red indicates high risk of bias.

**Table 4:** ROBINS‐I domain‐level judgements for principal outcomes of nonrandomized comparative studies.

ROBINS‐I judgements are result‐specific. One principal functional outcome contributing to the review was selected for each assessment.									
Study	Outcome/result assessed	D1	D2	D3	D4	D5	D6	D7	Overall
Chen et al.	IKDC score at 12 months	Serious	Moderate	Low	Moderate	Low	Serious	Moderate	Serious
Qureshi et al.	IKDC score at 3 months	Serious	Moderate	Low	Low	Low	Serious	Moderate	Serious
Magnussen et al.	IKDC score at 2 years	Serious	Moderate	Low	Low	Serious	Moderate	Moderate	Serious
Sözkesen et al.	Lysholm score at final clinical follow‐up (median, 12 months; range, 4–16 months)	Serious	Moderate	Low	Moderate	Low	Serious	Moderate	Serious
D1: confounding; D2: participant selection; D3: classification of interventions; D4: deviations from intended interventions; D5: missing data; D6: outcome measurement; D7: selection of the reported result.									
Judgement categories: Low, Moderate, Serious, Critical, or No information.									

*Note*: Green indicates low risk of bias, yellow indicates moderate risk of bias, red indicates serious risk of bias.

**Table 5:** Visual summary of risk of bias (RoB) 2 judgements for randomized controlled trials.

Study	RoB domains					
	D1	D2	D3	D4	D5	Overall
Ji et al.	Some concerns	Low risk	Some concerns	High risk	Some concerns	High risk
Cervellin et al.	Some concerns	Low risk	Low risk	Low risk	Some concerns	Some concerns
Gong et al.	Some concerns	Low risk	Low risk	High risk	Some concerns	High risk
Walters et al.	Low risk	Some concerns	Low risk	Low risk	Low risk	Some concerns
Rupreht et al.	Some concerns	Low risk	Some concerns	Low risk	Some concerns	Some concerns
Valentí Nin et al.	Some concerns	Low risk	Low risk	Low risk	Some concerns	Some concerns
Ye et al.	Low risk	Low risk	Low risk	High risk	Low risk	High risk
de Almeida et al.	Some concerns	Low risk	Some concerns	Low risk	Some concerns	Some concerns
Vadalà et al.	Some concerns	Low risk	Low risk	Low risk	Some concerns	Some concerns
Kumar et al.	Some concerns	Low risk	Low risk	High risk	Some concerns	High risk
Mirzatolooei et al.	Some concerns	Low risk	Low risk	Some concerns	Some concerns	Some concerns
Starantzis et al.	Some concerns	Some concerns	Some concerns	Low risk	Some concerns	Some concerns
Munde et al.	Low risk	Low risk	Low risk	Low risk	Low risk	Low risk

*Note*: Green indicates low RoB, yellow indicates some concerns, and red indicates high RoB. D1, bias arising from the randomization process; D2, bias due to deviations from intended interventions; D3, bias due to missing outcome data; D4, bias in measurement of the outcome; D5, bias in selection of the reported result.

**Table 6:** Visual summary of ROBINS‐I judgements for nonrandomized comparative studies.

Study	Risk of bias domains							
	D1	D2	D3	D4	D5	D6	D7	Overall
Chen et al.	Serious	Moderate	Low	Moderate	Low	Serious	Moderate	Serious
Qureshi et al.	Serious	Moderate	Low	Low	Low	Serious	Moderate	Serious
Magnussen et al.	Serious	Moderate	Low	Low	Serious	Moderate	Moderate	Serious
Sözkesen et al.	Serious	Moderate	Low	Moderate	Low	Serious	Moderate	Serious

*Note*: Green indicates low risk of bias, yellow indicates moderate risk of bias, red indicates serious risk of bias.

Among the randomized trials, four studies were judged to have high overall RoB, eight raised some concerns, and one was judged to have low overall RoB. The most frequent concerns involved insufficient reporting of allocation concealment, missing outcome data, lack of participant blinding for patient‐reported outcomes and absence of accessible prespecified statistical analysis plans. High overall RoB was primarily associated with the measurement of subjective patient‐reported outcomes in trials without adequate participant blinding.

All four nonrandomized comparative studies were judged to be at serious overall RoB. The main concerns involved residual confounding, historical or predictable allocation methods, participant selection, incomplete control of cointerventions, missing outcome data in one study and lack of blinding for patient‐reported outcomes.

### Certainty of evidence

Using the randomized‐trial evidence, the certainty of evidence for the meta‐analyzed patient‐reported outcomes ranged from very low to low. Certainty was very low for IKDC at 3 months, Lysholm at 3 and 6 months, and pain assessed using the VAS at approximately 12 months. Certainty was low for IKDC at 6 and 12 months and for Lysholm at 12 months and late post‐operative follow‐up. Downgrading reflected concerns related to RoB, unexplained inconsistency, indirectness of pain measurement and imprecision, depending on the outcome.

The late post‐operative Lysholm estimate was statistically different from zero but represented a clinically trivial difference and was considered fragile because it was based on only two trials and was almost entirely determined by one study. Formal assessment of publication bias was not possible because fewer than 10 studies contributed to each outcome. The complete GRADE assessment is presented in Table 7.

**Table 7:** GRADE assessment of certainty of evidence based on randomized controlled trials.

Outcome	RCTs/participants	Effect estimate (95% CI)	Risk of bias	Inconsistency	Indirectness	Imprecision	Publication bias	Certainty
IKDC score—3 months	3/212	MD 1.05 (−14.91 to 17.01)	Very serious^a^	Serious^b^	Not serious	Serious^c^	Not strongly suspected^d^	Very low (⊕◯◯◯)
IKDC score—6 months	3/221	MD 1.44 (−1.46 to 4.34)	Very serious^a^	Not serious	Not serious	Not serious^e^	Not strongly suspected^d^	Low (⊕⊕◯◯)
IKDC score—12 months	4/247	MD 2.65 (−1.83 to 7.13)	Very serious^a^	Not serious	Not serious	Not serious^e^	Not strongly suspected^d^	Low (⊕⊕◯◯)
Lysholm score—3 months	3/212	MD 1.58 (−12.44 to 15.60)	Very serious^f^	Serious^g^	Not serious	Serious^h^	Not strongly suspected^d^	Very low (⊕◯◯◯)
Lysholm score—6 months	3/251	MD 3.03 (−7.66 to 13.71)	Serious^i^	Serious^j^	Not serious	Serious^k^	Not strongly suspected^d^	Very low (⊕◯◯◯)
Lysholm score—12 months	3/203	MD −1.49 (−3.83 to 0.84)	Very serious^f^	Not serious	Not serious	Not serious^e^	Not strongly suspected^d^	Low (⊕⊕◯◯)
Lysholm score—late post‐operative follow‐up	2/91	MD 1.50 (1.22 to 1.77)	Serious^l^	Not serious	Not serious	Serious^m^	Not strongly suspected^d^	Low (⊕⊕◯◯)
VAS pain—approximately 12 months	3/120	MD −0.60 (−1.64 to 0.44)	Serious^n^	Not serious	Serious^o^	Serious^p^	Not strongly suspected^d^	Very low (⊕◯◯◯)

*Note*: GRADE ratings were based on the sensitivity analyses restricted to RCTs. The VAS analysis already included RCTs only. Clinical‐importance thresholds used for imprecision judgements were 9 points for IKDC, 10 points for Lysholm and 1.0 point for VAS on a 0–10 scale.

Abbreviations: CI, confidence interval; GRADE, Grading of Recommendations Assessment, Development and Evaluation; IKDC, International Knee Documentation Committee; RCT, randomized controlled trial; VAS, visual analogue scale.

^a^ Downgraded two levels for risk of bias because all or nearly all statistical weight came from trials judged at high risk of bias for subjective patient‐reported outcomes, principally because outcome measurement was not adequately blinded and additional concerns affected randomization, missing data, or selective reporting.

^b^ Downgraded one level for unexplained inconsistency because the individual estimates were discordant and statistical heterogeneity was substantial (*I* ^2^ = 77%).

^c^ Downgraded one level for imprecision because the 95% CI included both clinically important benefit and clinically important harm relative to the 9‐point IKDC threshold.

^d^ Formal assessment of publication bias was not possible because fewer than 10 studies contributed to each outcome. No additional evidence was identified that justified downgrading.

^e^ Not downgraded for imprecision because the entire 95% CI remained within the prespecified range for a clinically trivial effect.

^f^ Downgraded two levels for risk of bias because all included trials were judged at high risk of bias for the relevant subjective functional outcome.

^g^ Downgraded one level for unexplained inconsistency because effects differed in direction and statistical heterogeneity was substantial (*I* ^2^ = 65%).

^h^ Downgraded one level for imprecision because the 95% CI included clinically important benefit and harm relative to the 10‐point Lysholm threshold.

^i^ Downgraded one level for risk of bias because high‐risk trials contributed slightly more than half of the statistical weight, although one low‐risk trial also contributed substantially.

^j^ Downgraded one level for serious inconsistency because substantial unexplained heterogeneity was present (*I* ^2^ = 68%).

^k^ Downgraded one level for imprecision because the 95% CI included no effect and a clinically important benefit exceeding the 10‐point Lysholm threshold.

^l^ Downgraded one level for risk of bias because both trials had some concerns and the estimate was almost entirely determined by one trial.

^m^ Downgraded one level for imprecision/fragility because only 91 participants contributed and 99% of the weight came from one trial; the other trial reported extreme dispersion values and contributed almost no information, despite the narrow pooled CI.

^n^ Downgraded one level for risk of bias because the body of evidence included one high‐risk trial and two trials with some concerns for subjective pain outcomes.

^o^ Downgraded one level for indirectness because the studies assessed different pain constructs and anatomical contexts, including donor‐site pain, kneeling pain and general pain, rather than a single consistently defined pain outcome.

^p^ Downgraded one level for imprecision because the 95% CI included both no effect and a clinically important reduction in pain of at least 1 point.

### Meta‐analysis of functional outcomes

#### IKDC score

**Figure 2:**
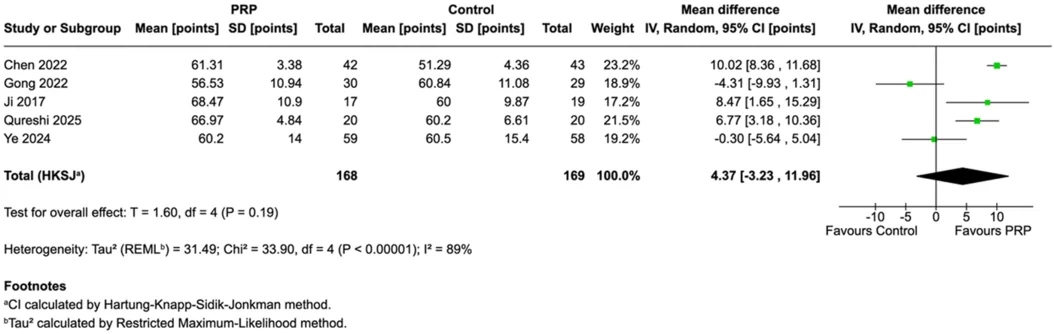
Forest plot of IKDC score (3 months). Meta‐analysis comparing PRP versus control therapies for IKDC score, measured at 3 months. The pooled effect did not reach statistical significance. IKDC, International Knee Documentation Committee; MD, mean difference; PRP, platelet‐rich plasma.

At 3 months, five studies involving 337 participants (168 in the PRP group and 169 in the control group) were included. No statistically significant between‐group difference was observed in IKDC score (MD, 4.37; 95% CI, −3.23 to 11.96; *p* = 0.19). Considerable heterogeneity was present (*I* ^2^ = 89%; *τ* ^2^ = 31.49) (Figure 2).

**Figure 3:**
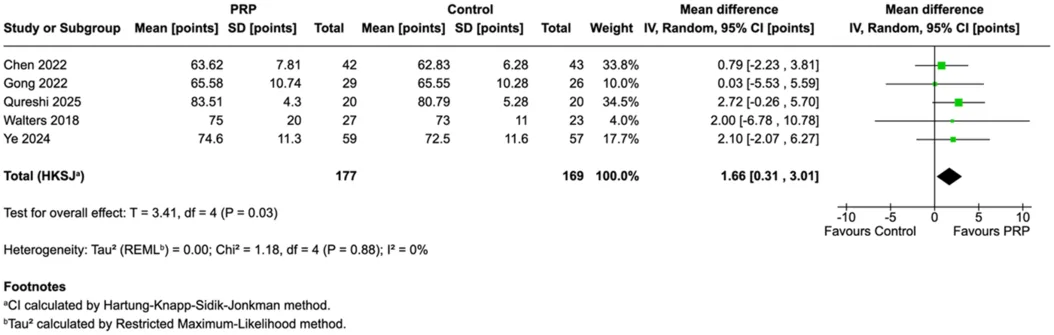
Forest plot of IKDC score (6 months). Meta‐analysis comparing PRP versus control therapies for IKDC score, measured at 6 months. The pooled effect did reach statistical significance. IKDC, International Knee Documentation Committee; MD, mean difference; PRP, platelet‐rich plasma.

At 6 months, five studies involving 346 participants (177 in the PRP group and 169 in the control group) demonstrated a small statistically significant difference favouring PRP (MD, 1.66; 95% CI, 0.31–3.01; *p* = 0.03), with no observed heterogeneity (*I* ^2^ = 0%; *τ* ^2^ = 0.00) (Figure 3).

**Figure 4:**
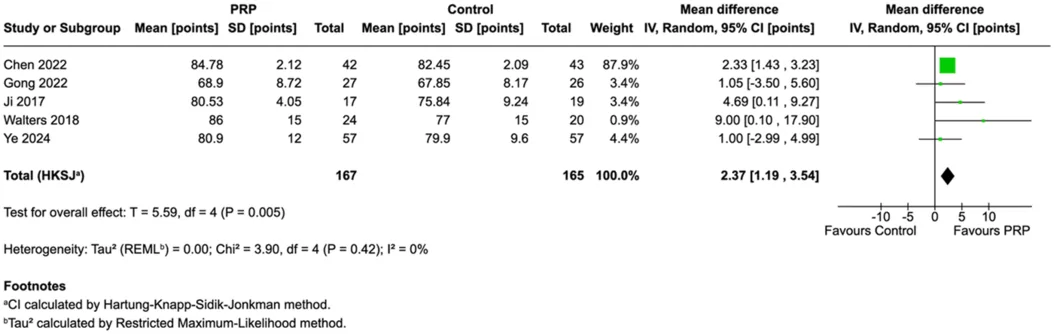
Forest plot of IKDC score (12 months). Meta‐analysis comparing PRP versus control therapies for IKDC score, measured at 12 months. The pooled effect did reach statistical significance. IKDC, International Knee Documentation Committee; MD, mean difference; PRP, platelet‐rich plasma.

At 12 months, five studies involving 332 participants (167 in the PRP group and 165 in the control group) also demonstrated a small statistically significant difference favouring PRP (MD, 2.37; 95% CI, 1.19–3.54; *p* = 0.005), with no observed heterogeneity (*I* ^2^ = 0%; *τ* ^2^ = 0.00) (Figure 4).

#### Lysholm score

**Figure 5:**
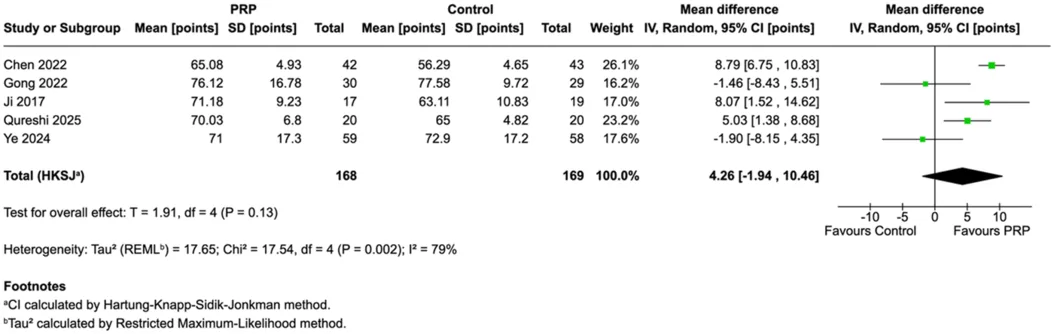
Forest plot of Lysholm score (3 months). Meta‐analysis comparing PRP versus control therapies for Lysholm score, measured at 3 months. The pooled effect did not reach statistical significance. MD, mean difference; PRP, platelet‐rich plasma.

At 3 months, five studies involving 337 participants (168 in the PRP group and 169 in the control group) showed no statistically significant between‐group difference in Lysholm score (MD, 4.26; 95% CI, −1.94 to 10.46; *p* = 0.13). Substantial heterogeneity was observed (*I* ^2^ = 79%; *τ* ^2^ = 17.65) (Figure 5).

**Figure 6:**
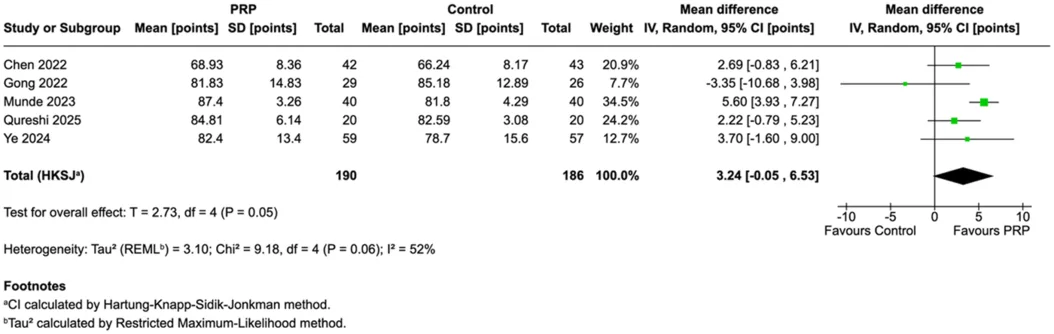
Forest plot of Lysholm score (6 months). Meta‐analysis comparing PRP versus control therapies for Lysholm score, measured at 6 months. The pooled effect did not reach statistical significance. MD, mean difference; PRP, platelet‐rich plasma.

At 6 months, five studies involving 376 participants (190 in the PRP group and 186 in the control group) demonstrated a borderline difference favouring PRP; however, the CI crossed the null value (MD, 3.24; 95% CI, −0.05 to 6.53; *p* = 0.05). Moderate heterogeneity was observed (*I* ^2^ = 52%; *τ* ^2^ = 3.10) (Figure 6).

**Figure 7:**
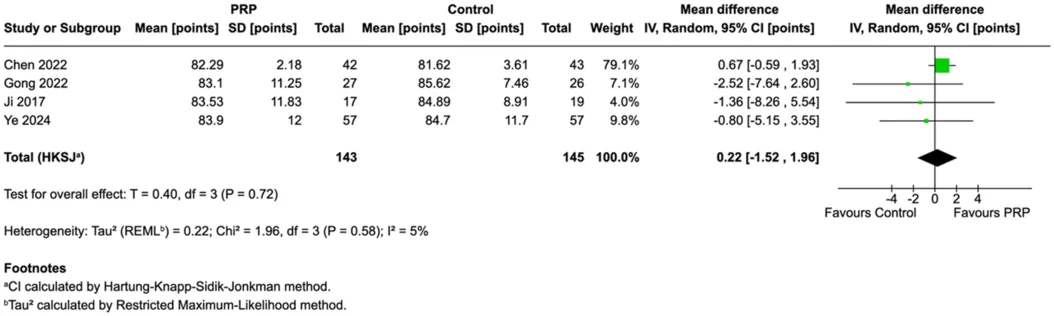
Forest plot of Lysholm score (12 months). Meta‐analysis comparing PRP versus control therapies for Lysholm score, measured at 12 months. The pooled effect did not reach statistical significance. MD, mean difference; PRP, platelet‐rich plasma.

At 12 months, four studies involving 288 participants (143 in the PRP group and 145 in the control group) showed no statistically significant between‐group difference (MD, 0.22; 95% CI, −1.52 to 1.96; *p* = 0.72), with minimal heterogeneity (*I* ^2^ = 5%; *τ* ^2^ = 0.22) (Figure 7).

**Figure 8:**
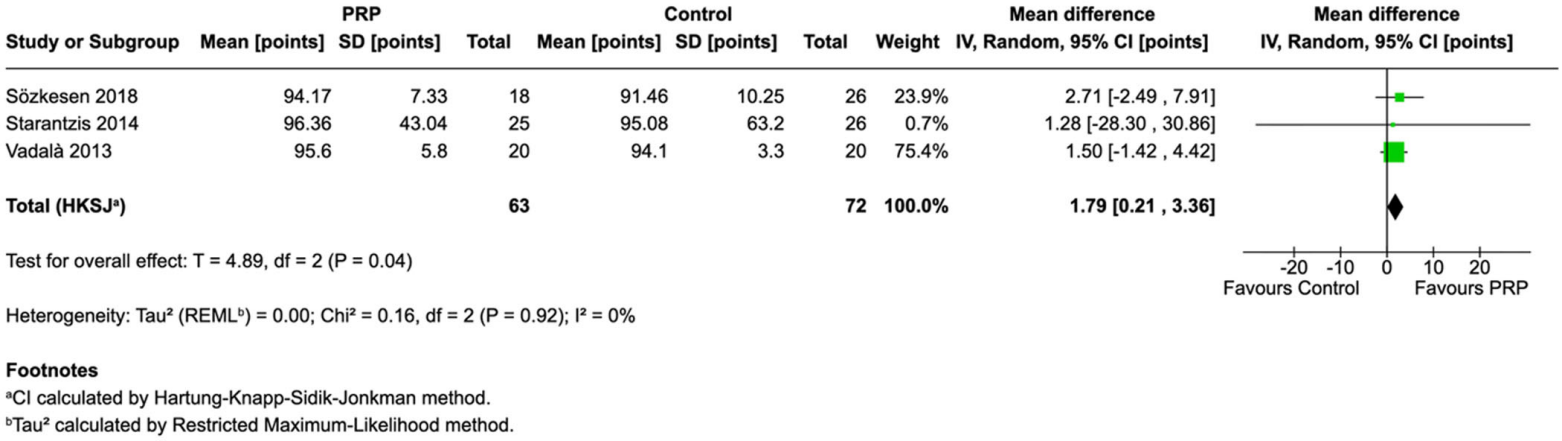
Forest plot of the Lysholm score at late post‐operative follow‐up. Meta‐analysis comparing platelet‐rich plasma (PRP) with control. CI, confidence interval; MD, mean difference.

At late post‐operative follow‐up, three studies involving 135 participants (63 in the PRP group and 72 in the control group) demonstrated a small statistically significant difference favouring PRP (MD, 1.79; 95% CI, 0.21–3.36; *p* = 0.04), with no observed heterogeneity (*I* ^2^ = 0%; *τ* ^2^ = 0.00) (Figure 8).

### Pain outcomes

**Figure 9:**
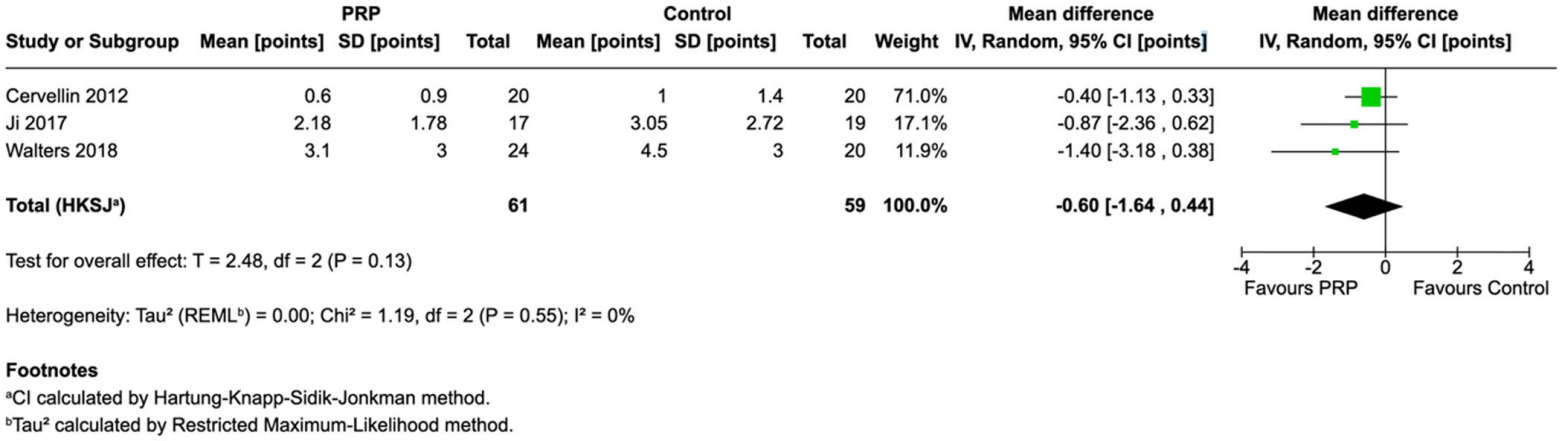
Forest plot of pain assessed using the visual analogue scale (VAS) at approximately 12 months. Meta‐analysis comparing platelet‐rich plasma (PRP) with control. Negative mean differences (MDs) favour PRP. CI, confidence interval.

Three RCTs involving 120 participants (61 in the PRP group and 59 in the control group) evaluated pain using the VAS at approximately 12 months. No statistically significant reduction in pain was observed with PRP compared with control (MD, −0.60; 95% CI, −1.64 to 0.44; *p* = 0.13), with no observed heterogeneity (*I* ^2^ = 0%; *τ* ^2^ = 0.00) (Figure 9).

#### Sensitivity analyses restricted to RCTs

Post hoc sensitivity analyses restricted to RCTs materially affected the interpretation of most functional outcomes. For the IKDC score, no statistically significant between‐group differences were observed at 3 months (MD, 1.05; 95% CI, −14.91 to 17.01; *p* = 0.80; *I* ^2^ = 77%), 6 months (MD, 1.44; 95% CI, −1.46 to 4.34; *p* = 0.17; *I* ^2^ = 0%) or 12 months (MD, 2.65; 95% CI, −1.83 to 7.13; *p* = 0.16; *I* ^2^ = 4%). Thus, the statistically significant differences observed in the primary analyses at 6 and 12 months were no longer statistically significant after exclusion of nonrandomized studies.

Similarly, no statistically significant between‐group differences were observed in Lysholm scores at 3 months (MD, 1.58; 95% CI, −12.44 to 15.60; *p* = 0.68; *I* ^2^ = 65%), 6 months (MD, 3.03; 95% CI, −7.66 to 13.71; *p* = 0.35; *I* ^2^ = 68%) or 12 months (MD, −1.49; 95% CI, −3.83 to 0.84; *p* = 0.11; *I* ^2^ = 0%).

At late post‐operative follow‐up, the difference favouring PRP remained statistically significant after restriction to two RCTs (MD, 1.50; 95% CI, 1.22–1.77; *p* = 0.009; *I* ^2^ = 0%). However, this estimate should be interpreted cautiously because it was based on only two trials, was predominantly influenced by one study and included unusually large dispersion estimates reported in the other trial.

A separate sensitivity analysis was not performed for the VAS outcome because all studies contributing to the primary VAS meta‐analysis were RCTs. The corresponding forest plots are presented in Figures S1–S7.

### Narrative synthesis

#### Objective knee stability (KT‐1000 arthrometer)

Several studies reported objective anterior knee stability using the KT‐1000 arthrometer [3, 19, 28, 31]. Although some individual studies described small, transient reductions in anterior laxity favouring PRP at early follow‐up, these findings were inconsistent and not sustained at mid‐ to long‐term follow‐up. Most studies did not demonstrate statistically significant or clinically meaningful differences between PRP and control groups. Due to heterogeneity in testing protocols, applied forces, assessment timing, graft types and reporting formats, quantitative synthesis was not feasible.

#### MRI‐based outcomes

MRI outcomes were synthesized narratively because of substantial heterogeneity in imaging protocols, outcome definitions and timing of assessment [1, 5, 6, 11, 14, 20, 24, 26, 29, 32, 34, 35]. Some studies reported early differences in graft signal intensity or appearance favouring PRP; however, these findings were inconsistent, method‐dependent and not sustained across later follow‐up intervals. Importantly, these imaging changes should be regarded as surrogate markers of uncertain clinical significance because they did not consistently correspond to improvements in patient‐reported function, activity level, pain or objective knee stability. Variability in MRI sequences, scoring systems and regions of interest further limited direct comparisons and precluded quantitative synthesis.

#### KOOS and Tegner scores

KOOS and Tegner activity scores were reported in a limited number of studies [11, 17, 28, 29, 31, 35]. Both outcomes improved over time in PRP and control groups, reflecting expected post‐operative recovery following ACLR. However, no consistent or clinically meaningful advantage favouring PRP was identified. Heterogeneity in outcome reporting, follow‐up intervals and limited data availability precluded meta‐analysis for these outcomes.

## DISCUSSION

The principal finding of this systematic review and meta‐analysis is that PRP augmentation during ACLR did not provide a consistent or robust improvement in patient‐reported knee function. Although the primary analyses combining randomized and nonrandomized studies identified small statistically significant differences favouring PRP for IKDC scores at 6 and 12 months and for the Lysholm score at late post‐operative follow‐up, the IKDC findings were no longer statistically significant when the analyses were restricted to RCTs. The late post‐operative Lysholm difference persisted in the RCT‐only analysis but was based on only two trials and should be considered fragile. No consistent advantages were demonstrated for pain, objective knee stability, activity level or long‐term functional recovery. Imaging‐based outcomes showed possible early differences in graft appearance; however, these findings were heterogeneous, inconsistent and of uncertain clinical relevance because they did not consistently correspond to better functional or stability outcomes.

### Comparison with previous evidence and added value of the present review

The findings of the present review are broadly consistent with previous systematic reviews and meta‐analyses evaluating PRP augmentation in ACLR [4, 7, 8, 18, 36]. These more recent evidence syntheses generally reported possible small improvements in selected patient‐reported outcomes, but no consistent or clinically meaningful benefits in objective knee stability, graft integration, or durable functional recovery.

Earlier systematic reviews primarily focused on imaging‐based graft maturation, graft–bone interface healing and ligamentization [2, 9, 33]. Although some individual studies suggested accelerated biological or imaging‐based healing, these reviews emphasized substantial heterogeneity in PRP preparation, application techniques, graft selection, imaging protocols and follow‐up intervals, preventing definitive conclusions regarding clinical efficacy.

The present results therefore reinforce, rather than overturn, the prevailing interpretation of the available evidence. The principal contribution of this review is a more current, time‐specific and methodologically conservative estimate of the magnitude and certainty of PRP‐related effects. The updated search incorporated recently published comparative studies, while separate analyses at 3, 6 and 12 months allowed the temporal pattern of patient‐reported outcomes to be examined. Furthermore, REML estimation combined with Hartung–Knapp–Sidik–Jonkman adjustments was used to account for uncertainty arising from the small number of studies included in most pooled analyses. The GRADE assessment also enabled the findings to be interpreted in the context of RoB, inconsistency and imprecision. In addition, the sensitivity analyses restricted to RCTs allowed the robustness of the pooled estimates to be examined according to study design and demonstrated that the statistically significant IKDC findings from the combined analyses were sensitive to the inclusion of nonrandomized evidence.

Importantly, although the primary analyses identified statistically significant differences in IKDC scores at 6 and 12 months, the pooled MDs of 1.66 and 2.37 points, respectively, were substantially below commonly reported minimal clinically important difference thresholds [22]. Furthermore, neither difference remained statistically significant after restriction to RCTs. Therefore, the inclusion of more recent evidence does not materially change the clinical interpretation of PRP augmentation. Rather, the findings emphasize both the distinction between statistical significance and patient‐perceived clinical benefit and the importance of study design when interpreting pooled estimates.

### Patient‐reported functional outcomes (IKDC and Lysholm scores)

In the primary analyses combining randomized and nonrandomized studies, small statistically significant differences favouring PRP were observed for IKDC scores at 6 and 12 months, whereas no significant difference was observed at 3 months. However, the 6‐ and 12‐month differences were no longer statistically significant after exclusion of the nonrandomized studies. This attenuation indicates that the combined estimates were sensitive to study design and may have been influenced by residual confounding in the observational evidence. Potential sources of confounding include graft choice, rehabilitation protocols, baseline activity level, concomitant procedures and surgeon preference.

Even in the primary analyses, the IKDC differences of 1.66 points at 6 months and 2.37 points at 12 months were substantially below commonly reported minimal clinically important difference thresholds of approximately 9 to 11 points. Therefore, neither the magnitude nor the robustness of these findings supports a clinically meaningful functional benefit of PRP for most patients.

For the Lysholm score, no statistically significant differences were observed at 3, 6 or 12 months. The 3‐month analysis showed substantial heterogeneity in both the primary and RCT‐only analyses, while restriction to randomized trials also resulted in wide CIs at 6 months, indicating inconsistency and limited precision. A small statistically significant difference remained at late post‐operative follow‐up in the RCT‐only analysis; however, this estimate was based on only two trials, was predominantly influenced by one study and included unusually large dispersion estimates reported in the other trial. Moreover, the difference of 1.50 points on a 0‐ to 100‐point scale was unlikely to represent an important clinical benefit. Overall, the functional findings do not demonstrate a consistent, robust, or clinically meaningful advantage of PRP augmentation.

### Activity level and knee‐related quality of life (Tegner and KOOS)

Tegner activity scale and KOOS outcomes improved over time in both PRP and control groups, reflecting the expected recovery trajectory following ACLR. However, no consistent or clinically meaningful differences favouring PRP were identified. These findings suggest that PRP does not appear to enhance return to higher activity levels or broader knee‐related quality of life beyond that achieved with standard surgical reconstruction and rehabilitation.

The absence of benefit in these outcomes may be explained by the multifactorial nature of functional recovery after ACLR, which depends not only on biological graft healing but also on neuromuscular control, psychological readiness, rehabilitation adherence and sport‐specific demands—factors unlikely to be substantially modified by PRP alone.

### Pain outcomes

The pooled analysis of pain assessed using the VAS at approximately 12 months included only RCTs and showed no statistically significant difference between PRP and control groups. The pooled MD was −0.60 points, with a CI crossing the null value and no observed heterogeneity. Because all contributing studies were randomized trials, a separate sensitivity analysis according to study design was not required.

These findings do not support a sustained analgesic benefit of PRP after ACLR. However, because the pooled analysis represented pain assessments at approximately 12 months, the result should not be interpreted as definitively excluding a transient effect during the immediate post‐operative period. Available data were insufficiently consistent to quantify pain separately at earlier follow‐up intervals.

### Objective knee stability (KT‐1000 arthrometer)

Objective knee stability assessed using the KT‐1000 arthrometer did not demonstrate consistent or clinically meaningful differences between PRP‐treated and control groups. While isolated studies reported small reductions in anterior tibial translation favouring PRP, these findings were inconsistent and not reproducible across different populations, graft types or follow‐up durations.

Variability in testing protocols, applied forces, timing of assessment and reporting formats further limited comparability and precluded quantitative synthesis. Overall, the available evidence does not demonstrate a measurable advantage of PRP in objective mechanical knee stability after ACLR.

### Imaging‐based outcomes

MRI‐based findings should be interpreted cautiously. Although some studies reported early reductions in graft signal intensity or a more homogeneous graft appearance in PRP‐treated groups, these observations were inconsistent and highly dependent on imaging protocols, MRI sequences, scoring systems and assessment timing.

Moreover, early imaging differences did not consistently translate into superior patient‐reported function, activity level, pain or objective knee stability. Accordingly, the available evidence does not establish that PRP‐related changes in MRI appearance represent a clinically meaningful acceleration of graft healing or ligamentization. These findings should therefore be regarded as exploratory surrogate outcomes rather than evidence of improved functional recovery or graft performance.

Standardized MRI protocols and studies specifically designed to evaluate the association between imaging findings and clinically relevant outcomes are required before such changes can be considered meaningful indicators of treatment benefit.

### Safety and complications

Across the included randomized and observational studies, PRP demonstrated a favourable safety profile. No serious adverse events, deep infections, graft failures attributable to PRP, or clinically relevant inflammatory reactions were reported. Minor complications, when described, were comparable between PRP and control groups and typically consisted of transient post‐operative discomfort [17, 32, 35].

However, interpretation of safety outcomes is limited by inconsistent reporting of adverse events, lack of standardized definitions and variability in follow‐up duration. Several studies did not prespecify complications as outcomes, raising the possibility of underreporting. Consequently, while current evidence suggests that PRP is safe in the context of ACLR, more rigorous and standardized safety reporting is warranted.

### Limitations

This review has several limitations. First, substantial heterogeneity and incomplete reporting of PRP preparation and application—including platelet concentration, leucocyte content, activation method, administered volume, preparation system, timing and site of application—precluded reliable subgroup analyses and prevented identification of an optimal PRP protocol. Although these variables were summarized descriptively at the study level, only three to five studies contributed to each pooled outcome and multiple protocol characteristics frequently differed simultaneously. Consequently, the independent influence of individual PRP characteristics could not be determined.

Second, the primary meta‐analyses combined RCTs and nonrandomized comparative studies. Although this approach allowed inclusion of the available comparative evidence, it may have increased susceptibility to residual confounding related to graft choice, rehabilitation protocols, baseline activity level, concomitant procedures and surgeon preference. The post hoc sensitivity analyses restricted to randomized trials materially attenuated the IKDC findings, indicating that the pooled estimates were sensitive to study design. However, relatively few randomized trials contributed to each sensitivity analysis, resulting in wide CIs and limited precision.

Third, the late post‐operative Lysholm sensitivity analysis was based on only two randomized trials, was almost entirely influenced by one study and included unusually large reported standard deviations in the other trial. This estimate should therefore be considered fragile and exploratory.

Fourth, several randomized trials were judged to have some concerns or a high RoB, whereas all nonrandomized comparative studies were judged at serious RoB, further reducing confidence in the pooled estimates. Fifth, data were extracted by one investigator and subsequently checked by a second investigator, rather than being independently extracted in duplicate. Although this verification process was intended to improve accuracy and completeness, it is less robust than fully independent duplicate extraction and may have increased the risk of extraction errors or reviewer‐dependent bias.

Sixth, two included trials enroled mixed adolescent and adult populations and age‐stratified outcome data were unavailable; therefore, minor indirectness related to participant age cannot be excluded.

Finally, imaging outcomes were synthesized narratively because of substantial methodological variability, including differences in MRI protocols, sequences, scoring systems and assessment timing. Most included studies also reported short‐ to mid‐term follow‐up, with limited data beyond 12 months. Consequently, the available evidence remains insufficient to determine whether PRP provides durable benefits in graft integration, long‐term knee function, objective stability or clinically relevant recovery. Future adequately powered RCTs using standardized PRP protocols, consistent outcome definitions and extended follow‐up are required.

## CONCLUSION

The available evidence does not demonstrate a consistent or robust clinically meaningful improvement in patient‐reported knee function, pain or objective stability with PRP augmentation after ACLR. Although small statistically significant differences favouring PRP were observed for selected functional outcomes in analyses combining randomized and nonrandomized studies, the IKDC findings at 6 and 12 months were no longer statistically significant when the analyses were restricted to RCTs. A small late post‐operative Lysholm difference persisted in the RCT‐only analysis, but this finding was based on only two trials and should be considered fragile. Imaging findings were heterogeneous and of uncertain clinical significance. Current evidence therefore does not support the routine use of PRP during or after ACLR.

## AUTHOR CONTRIBUTIONS

André Bosio Castro contributed to study conceptualization, data extraction, statistical analysis and drafting of the manuscript. Eiki Kobayashi contributed to study selection, data extraction and critical revision of the manuscript. Laura Azevedo contributed to methodological design and critical revision of the manuscript. Pedro Jorge contributed to study design, supervision, interpretation of results and final revision of the manuscript.

## FUNDING INFORMATION

The authors have no funding to report.

## ETHICS STATEMENT

The authors have nothing to report.

## CONFLICT OF INTEREST STATEMENT

The authors declare no conflicts of interest.

## Supporting information

Table S1. Platelet‐rich plasma preparation and application characteristics of the included studies.

Table S2. Complete RoB 2 signalling‐question assessments for randomized controlled trials. Supplementary Table S3. Complete ROBINS‐I signalling‐question assessments for nonrandomized comparative studies.

Figures S1–S7. Forest plots of the sensitivity analyses restricted to randomized controlled trials.

PRISMA 2020 Checklist. Completed Preferred Reporting Items for Systematic Reviews and Meta‐Analyses 2020 checklist indicating the location of each reporting item in the manuscript.

## Data Availability

All data generated or analyzed during this study are included in this published article and its [Link], [Link], [Link], [Link] files.
