# Supplementary material for: The effects of platelet‐rich plasma therapy on outcomes after anterior cruciate ligament reconstruction: A systematic review and meta‐analysis
Source: J Exp Orthop. 2026 Sep 21;13(3):e70913. doi: 10.1002/jeo2.70913 (PMC13592543; doi:10.1002/jeo2.70913)
Supplement: Supplementary file 2 [file JEO2-13-e70913-s002.docx]

**Supplementary Figures S1–S7**

*RCT-only sensitivity analyses*

Supplementary Figure S1. RCT-only sensitivity analysis for IKDC score at 3 months.

Supplementary Figure S2. RCT-only sensitivity analysis for IKDC score at 6 months.

Supplementary Figure S3. RCT-only sensitivity analysis for IKDC score at 12 months.

Supplementary Figure S4. RCT-only sensitivity analysis for Lysholm score at 3 months.

Supplementary Figure S5. RCT-only sensitivity analysis for Lysholm score at 6 months.

Supplementary Figure S6. RCT-only sensitivity analysis for Lysholm score at 12 months.

Supplementary Figure S7. RCT-only sensitivity analysis for Lysholm score at late postoperative follow-up.
